# Adaptive filtering of microseismic data for monitoring a water-conducting fractured zone in a mine

**DOI:** 10.1038/s41598-022-21441-8

**Published:** 2022-10-19

**Authors:** Hongjiang Li, Liang Han, Donglin Dong

**Affiliations:** 1 Department of Geological Engineering and Environment, China University of Mining and Technology, Beijing, 100083 China; 2 College of Safety Engineering, North China Institute of Science and Technology, Beijing, 101601 China

**Keywords:** Energy science and technology, Engineering, Physics

## Abstract

Water-conducting fractured zones in a rock mass can cause problems in mining. Attempts have been made to monitor their development using microseismic signals. However, due to the lack of prior information, it is difficult to filter out mixed low-frequency interference with traditional denoising methods. In this work, the proposed adaptive filtering algorithm is applied after the wavelet packets are decomposed. It is based on a cross-correlation analysis. The algorithm takes a high-quality signal in the common source waveform as prior information and applies the corresponding correlation coefficients between subbands as a threshold. The algorithm was verified with simulations. The results show that low-frequency interference can be effectively suppressed by filtering. For single-frequency interference, the signal-to-noise ratio increased from − 10.18 to 13.97, and the root-mean-square error was 43.88. For multi-frequency interference, it increased from − 10.01 and − 2.63 to 13.50 and 7.99. The root-mean-square errors were 46.31 and 138.07. The narrower the main frequency band of the interference signal and the less the overlap of the main frequency band of the interference signal and the effective signal, the better the filtering effect. When the algorithm was applied to microseismic data collected in the field, the number of effective channels increased and the accuracy improved. The development of a water-conducting fractured zone in the field was consistent with the microseismic location obtained after interference was removed by the algorithm, which indicates that it is feasible to monitor a water-conducting fractured zone by analyzing microseismic waveforms with the adaptive filtering algorithm.

## Introduction

A water-conducting fractured zone is the macroscopic manifestation of fractures due to the instability of a rock mass under the combined action of mining stress and water pressure. The development height of the water-conducting fracture zone is of great significance in the aspects of water prevention and control in coal mines, water retaining mining in coal mines, co-mining of coal and gas, and mining under water bodies. Micro-fractures are precursors to the formation of a water-conducting fractured zone. In recent years, many scholars have used microseismic signals caused by micro-fractures to monitor the formation, development, inoculation, and evolution of water-conducting fractured zones in a mine, achieving useful results^1–6^. Compared with the previous research methods on the development height of water-conducting fracture zone, the microseismic monitoring technology has the advantages of high visualization, flexible layout, four-dimensional monitoring, dynamic real-time, etc. This technology receives the vibration signal generated when the coal and rock mass break, analyzes the arrival time of the signal, calculates the location of the microseismic event using the positioning algorithm, and then calculates the energy level of the microseismic event through the energy algorithm to build a overburden fracture model, based on which to study the spatial distribution of the water-conducting fracture zone in coal mining. However, a signal measured in the field will inevitably contain noise and interference due to the complexity of the environment. Noise signals and interference signals are quite different from each other. Noise signals mainly come from inside the system, such as thermal noise, alternating noise, inductive noise, etc. Noise is characterized by low energy, high frequency, and strong randomness. It has a large impact on the time–frequency interpretation and picking up the waveform. In contrast, interference mainly comes from outside the system, such as electromagnetic interference from transmission lines or mechanical interference caused by drilling and electromechanical equipment^7,8^. Interference is characterized by high energy, low frequency, and a concentrated distribution, and it has a large impact on positioning the source and calculating the energy.

At present, research into removing noise from microseismic signals is mainly focused on transforming the monitoring signal to another domain through a mathematical transformation, which separates the noise from the effective signal. The noise is then removed based on a threshold. Finally, a denoised signal is obtained by an inverse transformation. According to the literature, there are four categories of denoising methods: methods based on time-varying median filters, methods based on a Fourier transform, methods based on a wavelet transform, and methods based on empirical mode decomposition.

For example, a time-varying median filter was applied in Ref.^9^ to attenuate noise. A method for suppressing random noise based on time-varying median filtering was proposed in Ref.^10^. That method is good at suppressing sharp pulses but can easily distort the signal if all data points are processed uniformly, especially when the noise is uniformly distributed.

Reference^11^ improved the resolution of seismic signals using a Fourier scale transform. Reference^12^ proposed a noise-reduction method based on a fractional Fourier transform. Such methods can improve the resolution of the signal, but they rely on the stationary statistical characteristics of the signal. When analyzing random non-stationary or non-linear signals with abrupt characteristics, such as seismic signals, they often fail to obtain satisfactory results.

To reduce the noise in non-stationary signals, such as microseismic signals, some scholars have used a wavelet transform or a wavelet packet transform to process and analyze the signal^13,14^. However, the selection of the wavelet basis for the signal decomposition is often affected by human subjective factors.

Thus, some scholars have proposed using empirical mode decomposition or the improved ensemble empirical mode decomposition^15,16^. In this method, a noisy signal is usually decomposed into the sum of multiple intrinsic mode functions. Some of these are discarded in an analysis of the signal characteristics. The remaining intrinsic mode functions are then reconstructed. For example, Ref.^17^ used empirical mode decomposition to selectively filter and eliminate noise according to the spectral characteristics of the decomposition of microseismic signals. Reference^18^ combined empirical mode decomposition and independent component analysis to reduce the noise in microseismic signals, achieving good results. However, these methods have obvious shortcomings, such as modal aliasing and the end-point effect, which lead to poor noise reduction.

Significantly, most of the above methods require prior knowledge about the features in the signal, but such prior knowledge is often unavailable in practice. For example, it is difficult to analyze a microseismic signal polluted by interference because there are many kinds of interference, the interference has a large amount of energy, and it is mainly located in a low-frequency band. Moreover, it is difficult to filter out interference in a microseismic signal with traditional denoising methods. During data analysis, the channels containing interference are usually directly eliminated, which can also lead to the underreporting of a considerable number of microseismic events, thus indirectly affecting the accuracy of coal and rock fracture analysis and energy statistics.

**Figure 1:**
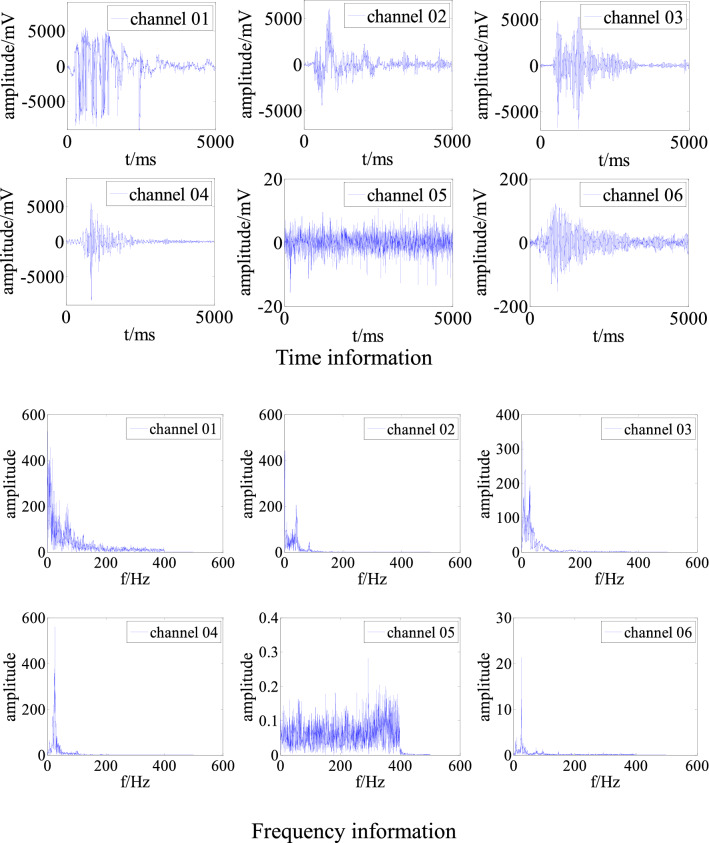
Multi-channel waveforms for a single event measured in the field.

Figure 1 shows typical multi-channel microseismic waveforms for a single event collected by the authors during the mining of a working face in Shandong Province, China. In these time- and frequency-domain graphs, the signal-to-noise ratio (S/N) for channels 01 and 02 is high, but the waveforms are distorted, indicating that the sensors were affected by interference from the surroundings. Channels 03 and 04 have normal waveforms. Channel 05 has no obvious waveform, and it contains bottom noise. The waveform envelope of channel 06 is clear, but the S/N is low, indicating that the sensor was far from the source and that only a small amount of the fracture event energy was received.

**Figure 2:**
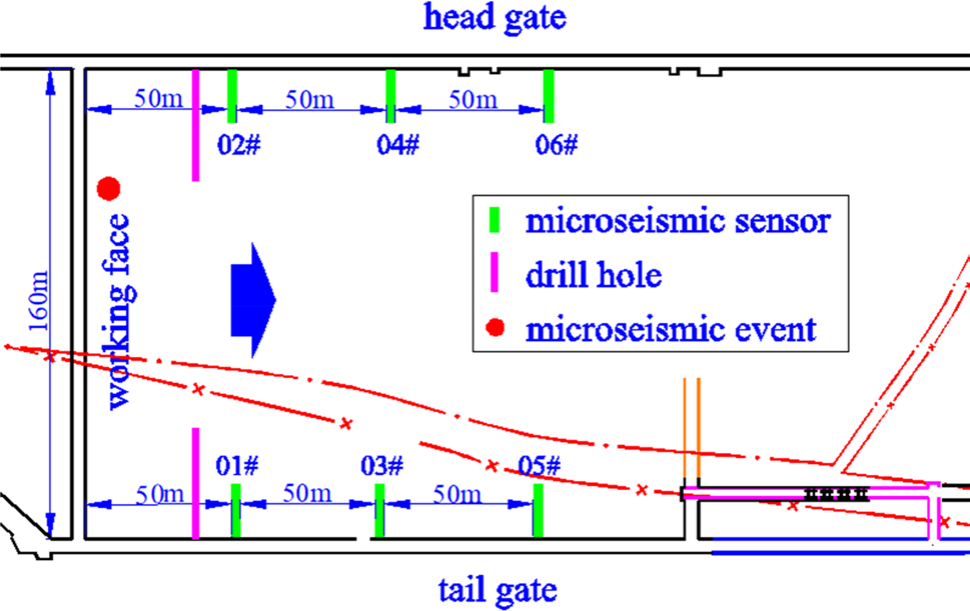
Site layout of microseismic sensors.

According to the on-site verification, as shown in Fig. 2, the microseismic event occurred at the mining face and there were obvious vibrations around it. There was also interference, due to drilling and grouting, as well as a strong electric current near sensors 01 and 02. Moreover, sensors 05 and 06 were relatively far from the vibration position. Due to the interference in channels 01 and 02, the microseismic event was not analyzed because there was not a good signal for the minimum number of effective channels required for positioning. According to the statistics for field data, similar microseismic events occur frequently.

To improve the signal quality and the accuracy of microseismic analysis and to understand the evolution of a water-conducting fractured zone in a mine, the authors use uncontaminated common source waveforms of microseismic events as prior information for wavelet packet decomposition and reconstruction. They apply self-adaptive filtering on the channels affected by the interference, and they explore an effective signal extraction method suitable for field application.

## Adaptive filtering

### Wavelet packet subband decomposition and reconstruction

Wavelet packet decomposition can decompose the non-decomposed high-frequency part of a decomposed wavelet into its high-frequency and low-frequency parts. If the minimum frequency of the signal is 0 and the maximum frequency is ω, then the signal frequency band is [0, ω] and the width of each subband is ω / 2^*n*^^19^.

Let *f* (*t*) be a time signal, and *p*^*i*^_*j*_ (*t*) represent the *i*th wavelet packet on layer *j*. *p*^*i*^_*j*_(*t*) is the wavelet packet coefficient. The algorithm for dyadic wavelet packet decomposition can be expressed as:

1 $$ \left\{ \begin{gathered} p_{0}^{1} (t) = f(t) \hfill \\ \ldots \hfill \\ p_{j}^{2i - 1} = \sum\limits_{k} {H(k - 2t)} p_{j - 1}^{i} (t) \hfill \\ p_{j}^{2i} = \sum\limits_{k} {G(k - 2t)} p_{j - 1}^{i} (t) \hfill \\ \end{gathered} \right. $$

The reconstruction algorithm can be expressed as:

2 $$ p_{j}^{i} (t) = 2\left[ {\sum\limits_{k} {h(t - 2k)p_{j + 1}^{2i - 1} (t)} + \sum\limits_{k} {g(t - 2k)p_{j + 1}^{2i} (t)} } \right] $$

In the above equations, *j* = *J-1*, *J-2*, …, 1, 0, $i = 2^{j} ,2^{j - 1} , \ldots ,2,1$, and $J = \log_{2} N$*.* Here, *G* and *H* are wavelet decomposition filters, and *h* and *g* are wavelet reconstruction filters. *h* and *H* are related to the scale function, whereas *G* and *g* are related to the wavelet function.

Wavelet packet decomposition produces detailed information about each frequency band of a polluted signal. The interference signal occurs only in some subbands. The expected signal can be filtered by reconstructing the remaining subbands with the help of a priori information.

### Cross-correlation analysis

In signal analysis, the cross-correlation function represents the degree of correlation between two time series, that is, it describes the random signals *x*(*t*) and *y*(*t*) at any two different times *t* and *t* + *τ*. The correlation degree between the signals is

3 $$ R_{xy} (\tau ) = \mathop \sum \limits_{t = - \infty }^{\infty } x(t)y(t + \tau ) $$

An analysis of measured data shows that the cross-correlation function between two common source waveforms has a maximum value, which is generally to the right of the midpoint of the time axis. The deviation time is the difference in the arrival times of the two waves. Therefore, to eliminate the influence of the arrival time difference between different channels on the cross-correlation analysis, the maximum value of the cross-correlation function is taken as the cross-correlation coefficient of the two waves.

If the cross-correlation coefficient is larger, more information between the signals is common to both. The cross-correlation coefficient has been used as a characteristic index to identify microseismic signals, blasting signals, and interference signals^20,21^. Thus, it is feasible to use the cross-correlation coefficient as a priori information to determine the weight of the interference in a subband.

### Adaptive filtering

**Figure 3:**
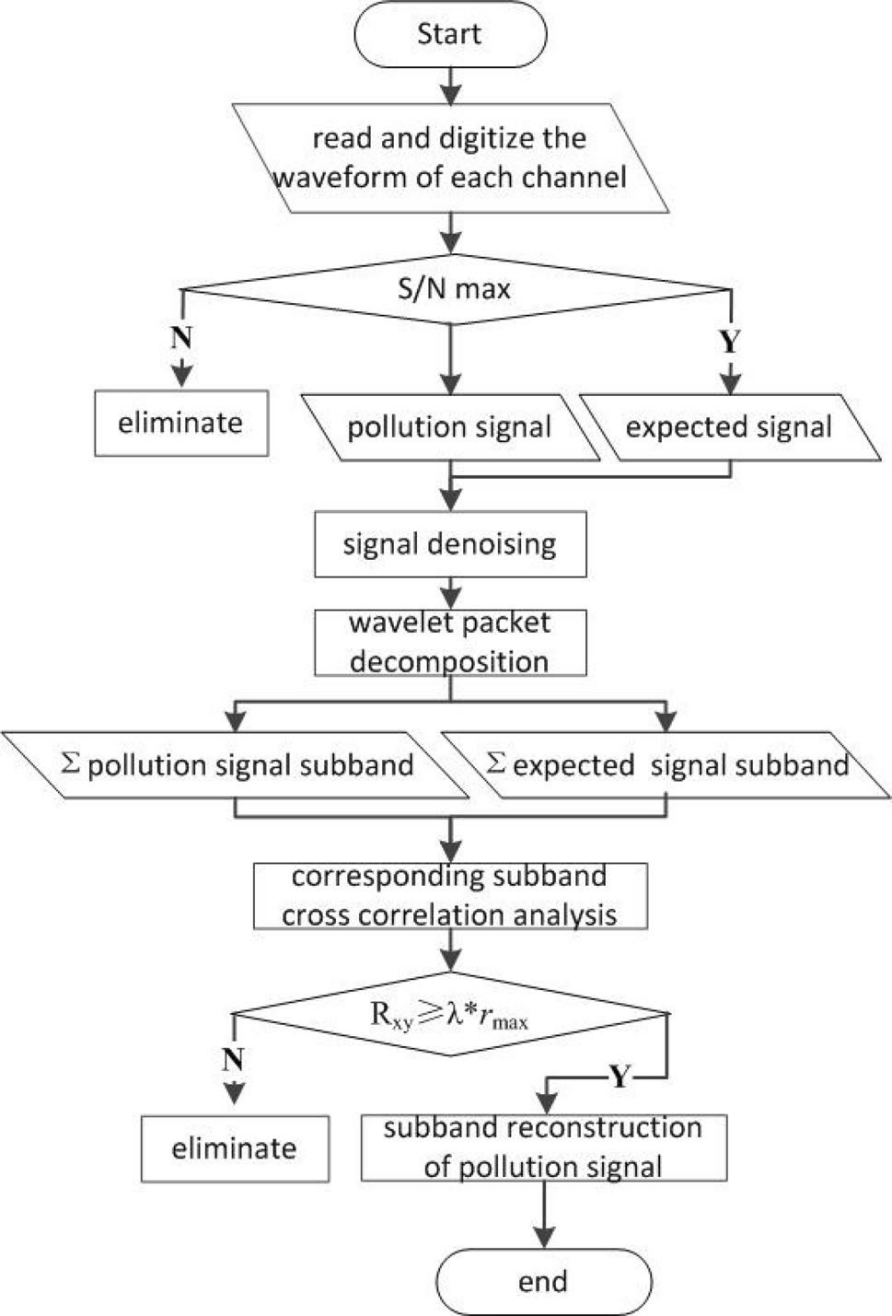
Flowchart for adaptive filtering.

Adaptive filtering can be summarized as extracting and determining the waveform with the highest similarity from the noise background. It can also be understood as a problem of waveform estimation. Assuming that the coal and rock mass comprise an isotropic medium, the common source waveform contains a significant amount of common information in the waveform characteristics of the same microseismic event^21^. Therefore, a channel with a relatively high waveform quality can be selected according to the statistical characteristics of the microseismic signal using indicators such as S/N. The denoised signal can be used as the expected signal in adaptive filtering. We follow the method given in Ref.^22^ of quantitatively describing the similarity of seismic waves by using the waveform correlation. After wavelet packet decomposition, the cross-correlation coefficient between the corresponding subbands of the interference signal and the expected signal can be used as the discrimination threshold. Subbands are discarded if the cross-correlation coefficient is lower than the threshold. The remaining subbands are reconstructed to obtain the adaptive filtered signal. The adaptive filtering process is shown in Fig. 3.

The specific process of adaptive filtering is as follows: first, read the waveforms of each channel in the same microseismic event, and select the channel with relatively high waveform quality as the expected signal of adaptive filtering according to the signal-to-noise ratio and other indicators; Secondly, the signal polluted by interference and the expected signal are decomposed by wavelet packet, and the cross-correlation coefficients between the corresponding subbands of the two are calculated. The ones with large correlation are retained, and the ones with small correlation are eliminated. Among them, the number of decomposition layers of wavelet packet, the selection of wavelet packet basis function and the value of cross-correlation coefficient need to be determined through data statistics and tests in specific engineering and simulation experiments. Finally, each subband of the processed pollution signal is reconstructed and the filtering is completed.

### Determination and discussion of the key parameters

#### Wavelet packet decomposition layers

If the sampling frequency is constant, the number of decomposition layers for a wavelet packet is equivalent to the bandwidth of the subband. Cao^23^ used the minimum working frequency of a microseismic sensor as the criterion. They recommended using a subband bandwidth of 15.625 Hz. The dominant frequency of the microseismic sensors used in a mine is 4.5 Hz, which means that the subband bandwidth should not be more than 4.5 Hz. The signals used for cross-correlation by the proposed algorithm are decomposed subbands. Theoretically, the finer the decomposition is, the more objective the description of the correlation is. Moreover, the low-frequency resolution should also be sufficient, as most of the interference is low frequency. However, using too many decomposition layers will slow down the calculation speed. For measured data, it is appropriate to decompose the wavelet packet into nine layers when the sampling frequency is 1000 Hz. In this situation, the subband bandwidth is 0.977 Hz.

#### Wavelet packet basis function

According to the theory, the basis function affects the signal processing. The basis function should be chosen by considering the error between the actual signal and the reconstructed signal. In this paper, through a large number of simulation experiments and tests, we chose the Sym8 wavelet packet basis as the basis function, as it has been verified as being more suitable for a non-stationary signal analysis^24^.

#### Discrimination threshold

The essence of the discrimination threshold is the cross-correlation coefficient, which is used to characterize the similarity between the corresponding subbands of the polluted signal and the expected signal. The waveform cross-correlation is affected by the difference in the arrival times, the amplitude, and the frequency. According to the statistics for waveforms^21^, the range of correlation coefficients between a rock-fracture waveform and an interference waveform is 0.02–0.14, whereas the range of correlation coefficients between different rock-fracture waveforms is 0.20–0.45. The correlation coefficient between different rock-fracture waveforms is higher in practice due to the non-limited signal length and frequency band range of the non-denoised waveform.

In addition, for waveforms obtained in different environments under different conditions, the statistical correlation coefficient will also change. Thus, it is more appropriate to use the relative value of the correlation coefficient to define the discrimination threshold. In this paper, the discrimination threshold is defined as:

4 $$\lambda =\frac{{r}_{i}}{{r}_{max}}$$

This discrimination threshold can be determined by limiting the distortion in a simulation experiment due to the test conditions and the number of decomposition layers of the wavelet packet. Under the conditions used in this paper, λ is in the range 0.75–0.85. *r*_*i*_ is the cross-correlation coefficient between the *i*th subband of the contaminated signal and the *i*th subband of the expected signal, and *r*_*max*_ is the maximum value of the cross-correlation coefficient in all subbands.

## Simulation experiments

### Experiment overview and objectives

**Figure 4:**
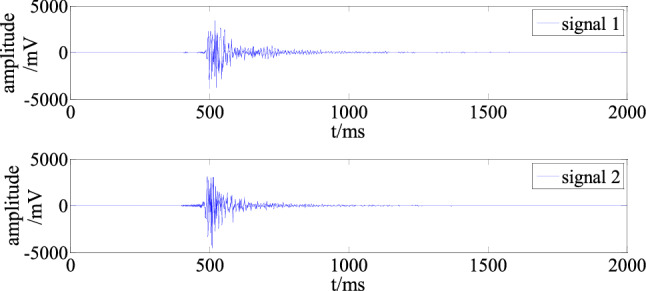
On-site measured microseismic waveforms.

The experimental signals were the waveforms measured by channels 03 and 04 for a microseismic event collected at 02:13:28 on June 27, 2021. The sampling frequency was 1000 Hz, and the number of sampling points was 5000. To highlight the main waveform, only the first 2000 sampling points were used. The signals for the two channels are shown in Fig. 4.

For the experiment, interference signals were constructed manually and measured on site. The authors of Ref.^7^ analyzed the time and frequency characteristics of different interference signals in mine microseisms. They found that the main frequency of an interference signal is usually about 14 Hz. Therefore, a sine wave with a frequency of 14 Hz was generated by MATLAB as the artificial interference source. Moreover, according to the statistics for a large number of on-site interference signals, the main frequency of the measured interference signals is mainly 0–20 Hz. Therefore, the invalid signal in the above frequency range can be selected as the measured interference source. In addition, to verify the applicability of the algorithm in other frequency bands, the artificial interference source in the main frequency range of the signal generated by MATLAB can also be used in experiments.

In the experiment, signal 1 was the desired signal. The interference signal was superimposed on signal 2 to construct the polluted signal affected by interference. The interference in the polluted signal was then filtered out with the proposed algorithm implemented in MATLAB to obtain the reconstructed signal 2'. According to the corresponding evaluation indexes, signals 2 and 2' were compared to test the effectiveness of the algorithm.

### Selection of evaluation indicators

In this paper, we use the S/N and root-mean-square error (RMSE)^25^, which are commonly used to evaluate the performance of signal denoising. The S/N before and after interference removal can be used to measure the denoising ability of the algorithm. The RMSE represents the deviation between the filtered signal and the original signal. It can reflect the distortion of the filtered signal to a certain extent.

The S/N is defined as:

5 $$\frac{S}{N}=10\mathrm{lg}\left(\frac{{\Sigma }_{i=1}^{\mathrm{\rm N}}{{x}_{\left(i\right)}}^{2}}{{\Sigma }_{i=1}^{\mathrm{N}}{\left({x}_{i}-{y}_{i}\right)}^{2}}\right)$$

The RMSE is defined as:

6 $$ {\text{RMSE}} = \sqrt {\frac{1}{N}\mathop \sum \limits_{i = 1}^{N} (x(i) - y(i))^{2} } $$

where *x*(*i*) is the original signal. *y*(*i*) is the polluted signal when the S/N is calculated before interference is removed, whereas it is the filtered signal when calculating the S/N and RMSE after interference removal.

### Simulation of removal of artificial single-frequency interference

**Figure 5:**
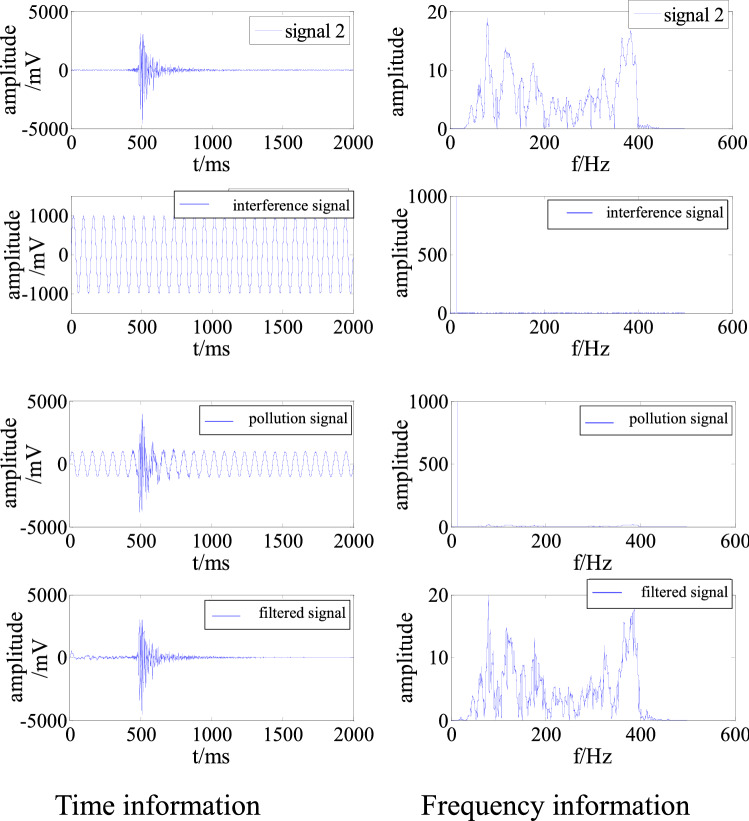
Time and frequency information for a signal polluted by single-frequency interference before and after filtering.

A simulation signal polluted by single-frequency interference was generated by superimposing the artificially constructed interference signal with a main frequency of 14 Hz and an amplitude of 1000 mV onto signal 2. According to the filtering parameters determined in the section on the “determination and discussion of the key parameters,” the signal was cleaned and denoised to obtain the time and frequency information, as shown in Fig. 5.

Figure 5 shows that the 14 Hz interference in the polluted signal is effectively suppressed by the adaptive filtering algorithm. The time and frequency characteristics of the filtered signal are very close to those of the original signal 2. To quantitatively evaluate the filtering, the S/N and RMSE were calculated with Eqs. (5) and (6), as shown in Table 1. Before the interference was removed, the S/N was − 10.18, indicating that the energy of the interference signal was greater than that of the effective signal. Traditional denoising algorithms regard this type of interference as an effective signal that is difficult to fit. After the interference was removed, the S/N had increased to 13.97, demonstrating that the denoising was successful. The filtering can distort the signal due to the different proportions of the interference in each subband, as there is a gray area close to the discrimination threshold that is also filtered out. The RMSE was 43.88.

**Table 1:** Evaluation of denoising of single-frequency interference.

S/N		RMSE
Before removal	After removal	
− 10.18	13.97	43.88

### Simulation of removal of measured multi-frequency interference

**Figure 6:**
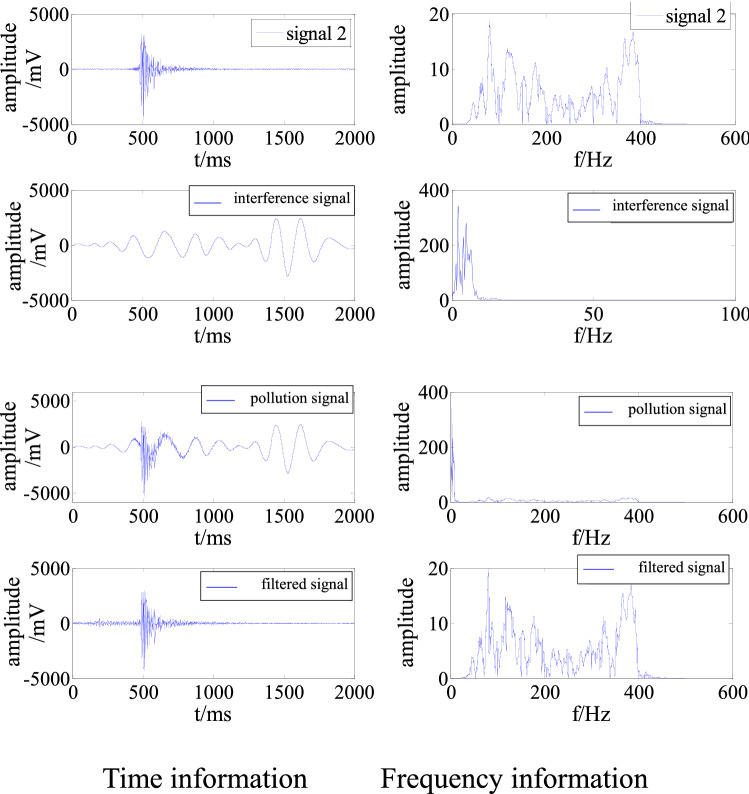
Time and frequency information of a signal polluted by multi-frequency interference before and after filtering.

To improve the universality of the simulation experiment, a measured low-frequency interference signal was used as the interference source. This signal was superimposed on signal 2 to generate a signal polluted by multiple frequencies. As above, the signal was cleaned and denoised to obtain the time and frequency information, as shown in Fig. 6.

Figure 6 demonstrates that the adaptive filtering algorithm effectively removed the low-frequency interference in the polluted signal so that the time and frequency characteristics of the signal are very close to those of the original signal 2. From Table 2, the S/N was − 10.01 before interference removal, indicating that the energy of the interference signal was greater than that of the effective signal. The S/N subsequently increased to 13.50. The RMSE was 46.31.

**Table 2:** Evaluation of denoising of multi-frequency interference.

S/N		RMSE
Before removal	After removal	
− 10.01	13.50	46.31

### Simulation of removal of artificial multi-frequency interference

**Figure 7:**
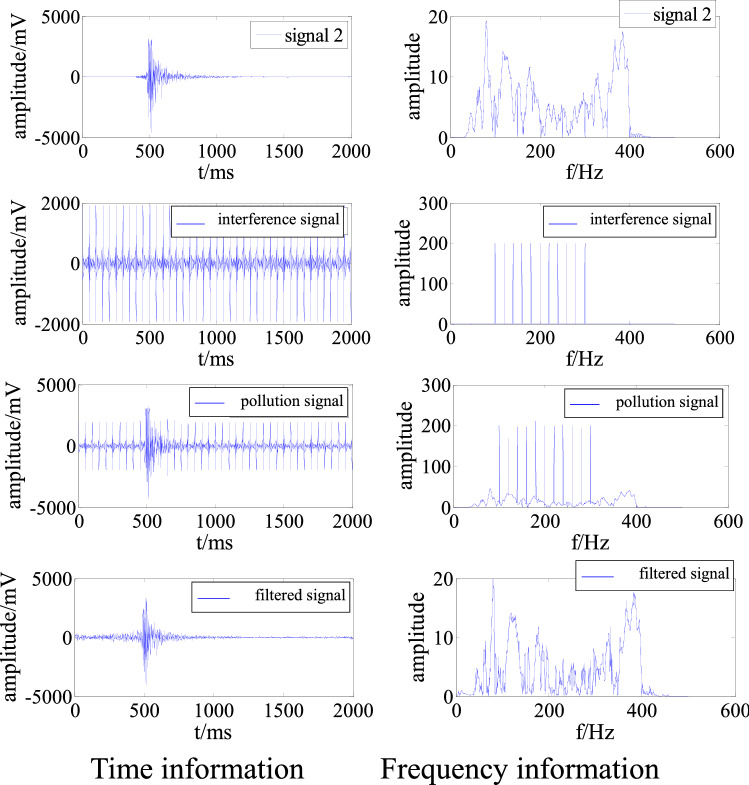
Time and frequency information of a signal polluted by multi-frequency interference before and after filtering.

In order to further verify the filtering effect of the adaptive filtering algorithm on interference signals in other frequency bands, a simulation signal polluted by multi frequency superposition is selected. Without losing generality, the main frequency of the interference source should overlap with the main frequency of signal2. Since it is difficult to collect such interference signals on site, manual construction is required. The frequency range of the interference signal is 100–300 Hz, the frequency interval is 20 Hz, and the amplitude is 200 mV. As above, the signal was cleaned and denoised to obtain the time and frequency information, as shown in Fig. 7.

Figure 7 demonstrates that the adaptive filtering algorithm effectively removed the interference overlapping with the main frequency of signal2 in the polluted signal so that the time and frequency characteristics of the signal are very close to those of the original signal 2. From Table 3, the S/N was − 2.63 before interference removal, indicating that the energy of the interference signal was greater than that of the effective signal. The S/N subsequently increased to 7.99. The RMSE was 138.07.

**Table 3:** Evaluation of denoising of multi-frequency interference.

S/N		RMSE
Before removal	After removal	
− 2.63	7.99	138.07

### Conclusion for the simulation experiment

The simulation results clearly show that the adaptive filtering algorithm was effective in filtering out interference signals. Removal of single-frequency artificial interference was more effective than removal of multi-frequency measured interference, which was more effective than removal of multi-frequency artificial interference. A comprehensive analysis suggests the possible reasons given below.

The narrower the main frequency band of the interference signal was, the better the filtering effect was.

The less the overlap between the main frequency band of the interference signal and the main frequency band of the effective signal was, the better the filtering effect was.

In the field, the main frequency band of the interference signal is usually very narrow, and most of the interference is concentrated in the low-frequency band. Thus, the overlap with the main frequency of the effective signal is small. The interference signal can be suppressed by this method. For a small part of the high-frequency noise, a suitable filter can be selected for denoising by acquiring a priori knowledge about the signal. Moreover, since the energy is very low, it has little impact on the source location and energy calculation.

## Field application

**Figure 8:**
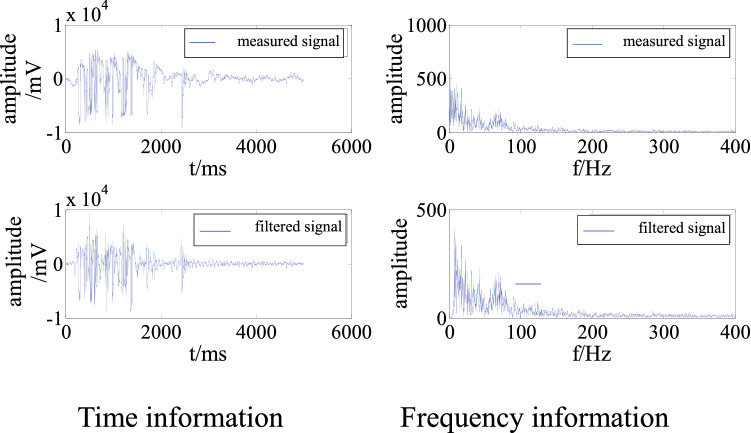
Time and frequency information for channel 01 before and after filtering.

**Figure 9:**
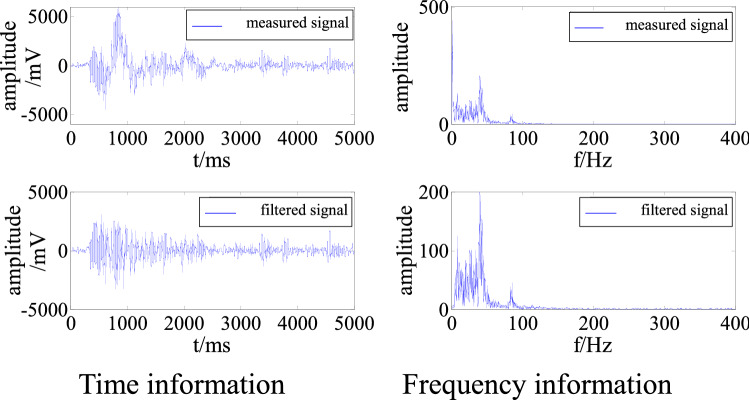
Time and frequency information for channel 02 before and after filtering.

The adaptive filtering algorithm was applied in an analysis of the on-site microseismic data shown in Fig. 1. This microseismic event was collected at 07:33:59 on June 29, 2021. We selected channel 04, which has a high waveform quality, as the expected signal and channels 01 and 02 as the polluted signals. The time and frequency information of the signals before and after adaptive filtering are shown in Figs. 8 and 9.

Figures 8 and 9 show that the adaptive filtering algorithm effectively filtered out the low-frequency interference from the polluted on-site signals. After filtering, the times of arrival of the signals in channels 01 and 02 was determined more accurately, so that they meet the conditions for source positioning. Moreover, the calculated energy was closer to the actual energy. More importantly, the microseismic event, which was originally rejected, can be used for microseismic positioning and statistical analysis, which would improve the accuracy of an on-site microseismic analysis.

**Figure 10:**
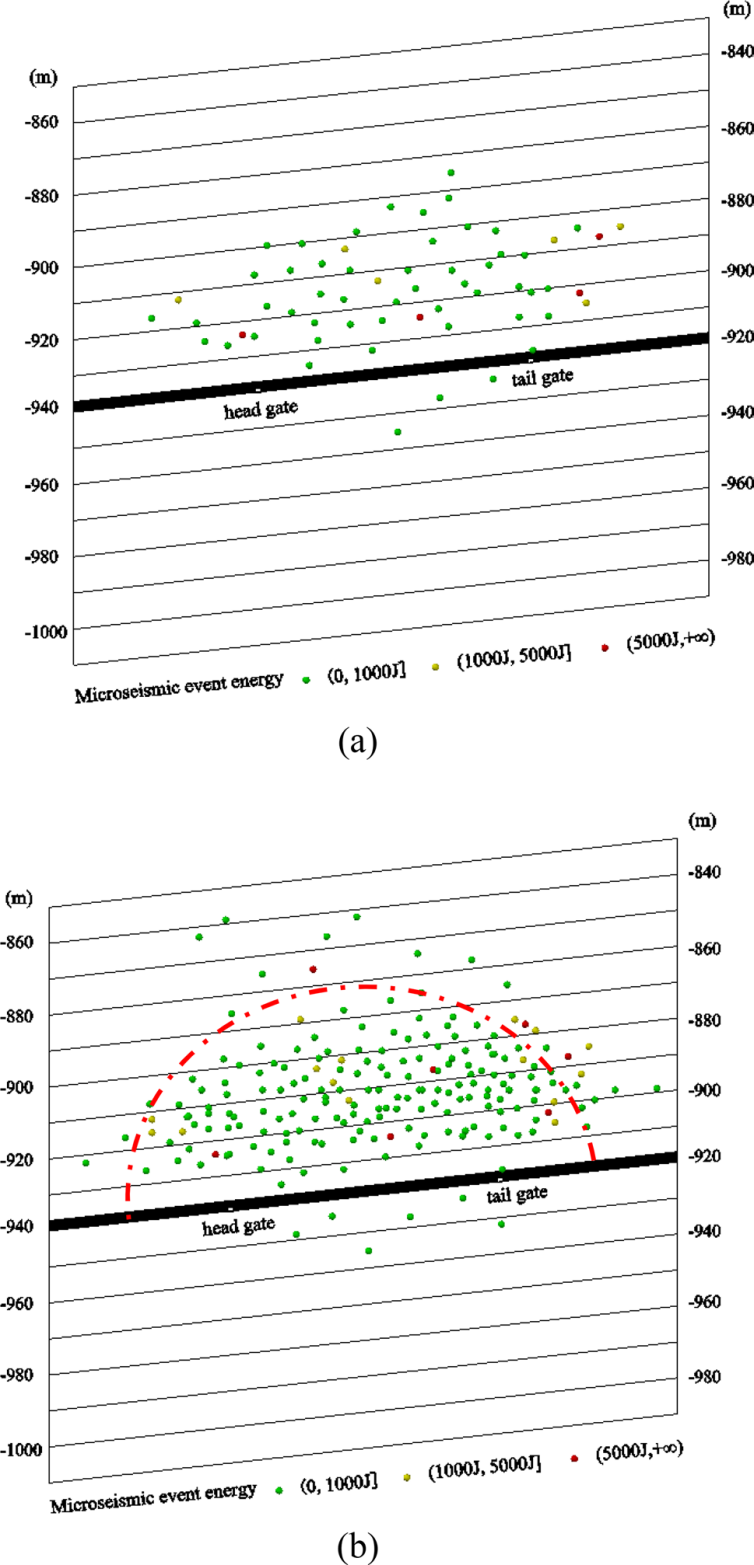
Vertical distributions of microseismic events (**a**) before and (**b**) after disturbance removal.

The positions of various microseismic events obtained after analysis were projected onto the vertical section of the working face, as shown in Fig. 10. The red dots, yellow dots and green dots represent the fracture point of the surrounding rock located by the microseismic system, in which red represents high energy microseismic events, yellow represents medium energy microseismic events, and green represents low energy microseismic events, as shown by the energy scale in the figure. Before interference removal, only 64 microseismic events could be projected onto the vertical profile. Of these, four high-energy microseismic events (red dots in Fig. 10a) were vertically distributed within 60 m above the coal seam (− 880 to − 940). After the interference was removed, 171 microseismic events could be projected onto the vertical profile. Further, the number of microseismic events meeting the focal location conditions increased significantly. Seven high-energy microseismic events (red dots in Fig. 10b) were vertically distributed within 80 m above the coal seam (− 860 to − 940). The length of the on-site working face was about 160 m. According to the estimate of the maximum fracture height of the overlying strata under full mining, the fracture height of 80 m, which was determined after the interference was removed, is more consistent with the actual situation on site. This demonstrates that the accuracy of microseismic location and the statistical analysis were greatly enhanced after interference was removed.

As shown in Fig. 10b, most of the microseismic events were within 60 m above the coal seam (− 880 to − 940). There were fewer microseismic events 60–80 m above the coal seam (− 860 to − 880), and their regularity was poor. This shows that the overburden in this layer had hardly been damaged and was not suitable for forming fractures. In addition, the medium- and high-energy microseismic events (yellow and red dots in the figure) were also mainly concentrated within 60 m above the coal seam (− 880 to − 940). Therefore, it was determined that the maximum development height of the water-conducting fractured zone was about 60 m above the coal seam.

According to actual measurements, the height of the collapse zone of the working face was 41 m, and the development height of the water-conducting fractured zone was 62 m. The microseismic location obtained after removing interference was consistent with the on-site measurements. This shows that the adaptive filtering algorithm proposed in this paper can improve the correction of a microseismic waveform as well as the source location and statistics of microseismic events. It provides stronger support for using a microseismic monitoring system to assess a water-conducting fractured zone.

## Conclusion

The proposed adaptive filtering algorithm is applied after the wavelet packets are decomposed. It is based on a cross-correlation analysis. The algorithm takes the high-quality signal of a common source waveform as prior information, and it also applies the corresponding correlation coefficients between subbands as a threshold.

The results show that low-frequency interference can be effectively filtered out under different conditions. For single-frequency interference, the S/N ratio increased from − 10.18 to 13.97, and the RMSE was 43.88. For multi-frequency interference, it increased from − 10.01 and − 2.63 to 13.50 and 7.99, the RMSE values were 46.31 and 138.07.

The characteristics of an interference signal have a direct impact on the filtering. Generally, the narrower the main frequency band of the interference signal and the shorter the overlap of the main frequency band of the interference signal and the effective signal are, the better the filtering effect is. The main frequency band of an on-site interference signal is usually very narrow, most of the interference is concentrated in the low-frequency band, and the overlap with the main frequency of the effective signal is small. Thus, the interference signal can be suppressed by this method.

When the algorithm was applied to on-site microseismic data, the times of arrival of the previously disturbed channel signals were determined more accurately. They met the conditions for source location, and the calculated energy was closer to the actual energy. The number of effective channels increased, and the accuracy of the analysis had improved.

The development of the water-conducting fractured zone measured in the field was consistent with the microseismic location obtained after interference was removed with the algorithm, which indicates that it is feasible to monitor a water-conducting fractured zone by analyzing microseismic waveforms with the adaptive filtering algorithm.

## Data Availability

The datasets generated and/or analysed during the current study are not publicly available due [the reasons agreed in the on-site project contract, the data can not be shared, because the publication and award application of other achievements are involved later] but are available from the corresponding author on reasonable request.
